# Conversations upon the Physical and Mental Hygiene of Girlhood

**Published:** 1881-02-20

**Authors:** T. S. Powell

**Affiliations:** Atlanta; Professor of Obstetrics and Diseases of Women, and Lecturer on Medical Ethics in the Southern Medical College


### Southern Medical Record.

EDITORS:

T. S. POWELL, M.D. W. T. GOLDSMITH, M.D. R. C. WORD, M.D.

*R. C. WORD, M.D., Managing Editor.*

☞All Communications and Letters on Business connected with the RECORD must be addressed to the Managing Editor.

VOL. XI. ATLANTA, GA., FEBRUARY 20, 1881. NO. 2.

### ORIGINAL AND SELECTED ARTICLES.

### CONVERSATIONS UPON THE PHYSICAL AND MENTAL HYGIENE OF GIRLHOOD.

BY T. S. POWELL, M. D.,

Professor of Obstetrics and Diseases of Women, and Lecturer on Medical Ethics in the Southern Medical College.

### CONVERSATION II.

Doctor—"Well, madam, I think it has been about ten days since my last visit. What have you to report this morning concerning Miss Mary's health? Something good, I know, judging from your eyes."

Mother—"Yes, Doctor. It gives me great pleasure to say that Mary has improved in every respect since I saw you last. She has obeyed your instructions to the letter. Her appetite has increased, and she certainly seems stronger, and also looks much better. I regret you cannot see her this morning, as she has just gone to ride with one of her school-mates."

Doctor—"From your report it is not very important, and I can call again in a few days."

Mother—"If you please. But, Doctor, I cannot let you go yet; I must ask you a few more questions."

Doc or—"Madam, as I teach my class that civility is due to a lady, however great the provocation may be, pressing as my engagements are, I will say I am yours to command, for a short while, at least."

Mother—"You are so kind. Walk into the parlor; do, Doctor, take that easy chair."

Doctor—"Thank you, madam. This is indeed a delightful chair —almost any wayworn doctor, so comfortably situated as I am, could make himself at least agreeable if not instructive. You can proceed, madam."

Mother—"I will Doctor, at once, as I know your time is precious. In reply to your last remark, in our first conversation, I am well satisfied that you are right, and think I can see why it is necessary to have sound, nourishing food in proper quantity, and sufficient exercise of the right kind, besides mental and physical rest, in order to make a perfect man or woman. I believe the Creator designed men and women to attain the utmost of human perfection, physically and mentally, as well as morally; and if that is His design, He certainly furnishes the means for its accomplishment."

Doctor—"You are right, madam. It is not my opinion that God intended any children to be born idiots or monsters, no more than He desires that the evil one should reign over the affections and passions of men."

Mother—"Neither do I believe, Doctor, that God makes children, men and women ugly and unlovable, or entails upon them any hereditary and loathsome disease."

Doctor—"You are again right, madam. All parents, who are themselves sound in mind and body, have it in their power to make their children handsome, intelligent, strong and healthy, consequently amiable, cheerful and happy. And these hereditary diseases, mental and physical, are the sins visited upon the children, even to the third and fourth generations—not sent upon them, though, by a spirit of divine vengeance, as some enemies of Christianity would argue when they wish to charge the Creator with being unjust and unmerciful, but they follow as a natural sequence in the economy of nature's laws, and one or both of the parents, or grand-parents of the children, have violated these laws, and they are, therefore, the real authors of these dreadful visitations upon innocent and helpless offspring."

Mother—"Yes, Doctor, I believe that to be the true solution of such sad misfortunes, and I wish to get your views upon these questions more fully at some future time, as I have several daughters younger than Mary, and I wish to guard them carefully from getting into her present state of ill health, and also to train them so far as I possibly can to become perfect women in every respect. I wish to ask you, just now, before you leave, about the use of vinegar as an article of diet. Do you know some people advised Mary to drink vinegar, and as much as she wanted; and a number of girls at school have persuaded Lucy, my next daughter, about thirteen years old, to use a great deal of vinegar. What do you think of such advice?"

Doctor—"I think it decidedly bad, when used as girls are in the habit of taking it. Pure apple vinegar, in moderation, as a condiment, acts beneficially upon some persons. There are certain conditions of the stomach when a small quantity of a pure vegetable acid prevents flatulence and fermentation of certain vegetable substances that have been eaten. But in a morbid or disordered state of the system, as is the case with your daughter Mary, vinegar is not only useless but destructive to health."

Mother—"From my own experience I know that vinegar, and all the native vegetable acids, should be used with caution, and with a knowledge of their effects in different conditions of the system, and a consideration of the articles of food with which we use the acid, I have noticed that when I take vinegar with certain kinds of food, great disorder of the stomach, with severe headache, ensues; while at other times, especially with vegetables intended to be eaten without being cooked, the vinegar I used with them seemed to increase my digestive powers."

Doctor—"Yes, madam, it certainly does produce the effects you have experienced; at least, if the vinegar does not increase the powers of digestion, it causes the food you have taken with it to assimilate more readily. It is upon this chemical principle that many persons, if in good health, can indulge with impunity in eating the richest soup, if they add to it a certain quantity of lemon juice; and upon the same principle many persons, especially fashionable ladies, are fond of apple sauce with their fresh meats. Thus you will perceive that pure vinegar is good in its proper place, like all the condiments, such as pepper, nutmeg, ginger, etc., but the dyspeptic should use any and all of these with great caution. A person in ill health may seem to be benefitted for a time by taking an unnatural stimulant to give a temporary increase of appetite, but great and lasting harm really ensues, because the artificial stimulant slowly destroys the tone of the stomach instead of restoring it to its normal capacity."

Mother—"I thank you, Doctor, I feel sure you are correct; do you think lard, butter and oil are wholesome, in or with our food?"

Doctor—"Much lard, especially such as you usually buy in the market, is very hurtful to dyspeptics. Genuine fresh butter, in moderation, and used at the table, does no harm but is beneficial. It is said by some that melted butter taken with the food is the most injurious."

Mother—"Yes, I have often heard that said, but of all unwholesome dishes, I think that meats fried in hog's lard are the most hurtful of any food, especially if eaten at supper."

Doctor—"You are right, madam; fried bacon and fried meats of any kind are not easily digested, and are not a suitable or wholesome food for women and children and men of sedentary habits. This is especially true of the meat on the market of late years, and commonly called bulk meat. When we consider how often it is packed and re-packed, shipped and re-shipped, and carelessly handled before it is at last eaten, and besides, often a large number of the hogs it was taken from were really diseased when they were killed, some of them probably with that fatal disorder trichiniasis, can we be surprised that it is not wholesome—that its effects are often serious when consumed in enormous quantities year after year, by the great majority of the people?"

Mother—"Yes, I have no doubt that the staple food of nearly all the working people in towns and cities is fried bulk meat, with the unwholesome corn meal of which you spoke in our last conversation."

Doctor.—"That is true, and the most stubborn, unyielding cases of diseases I have found among people who eat a great deal of this meat and other food reeking with the oil of hog's lard."

Mother.—"I am satisfied, Doctor, that the meat we buy is not as sound and wholesome as that we used to get from our father's smoke-house. Oh! how I long for those good old times to come again. Every one knows that cabbage is considered a very unwholesome vegetable, but I have fully tested its effects and will tell you my experience with it; I can eat cabbage in what you call cold slaw, and feel no inconvenience from it. I can also have it boiled perfectly tender in *clear water*, then chop it finely, add a little salt, a very little fresh butter and a few spoonsful of cream or sweet milk, then smother it for a few moments in a covered vessel, and I can eat it moderately warm and feel perfectly well afterwards; but if I have the cabbage boiled with bacon, bulk meat, or even good sweet ham, I cannot readily digest it, and I have learned from these tests that the cabbage, if fresh and sound, is not itself unwholesome, but the strong grease cooked with it and some other vegetables I could name, is what makes me sick, and so often brings on dyspepsia. I have also noticed that when cabbage is prepared in this way (without any meat), it has a more delightful flavor and is more delicious in every way."

Doctor.—"I thank you, madam, for your valuable experience. I have a very dear dyspeptic friend whose experience is precisely the same. He thinks the flavor is not then smothered by the strong and often rancid odor and taste of pork grease. We know that many articles of food, delicious and wholesome in themselves, are made unpalatable and injurious to health by the manner in which they are prepared for the table."

Mother—"Yes, I am confident of that. I believe I have as good digestion as the average women in good health, but I am satisfied that I cannot eat fried meats of any kind without affecting my health unpleasantly. I also do not feel quite well after eating ham or fresh pork, even when it has been thoroughly baked or boiled, unless I wait for it to become perfectly cold."

Doctor—"And that is really the most wholesome way in which it can be eaten. When taken into the stomach warm or hot the oil, in the fat of the meat is not easily digested, and no one but a strong, vigorous man of active, outdoor employment, can eat it thus with anything like impunity, even once a day. It is better for every one to refrain from eating warm fat pork at breakfast or supper."

Mother—"I am sure it is better. While the same kind of meat I eat at dinner does not hurt me at all. I find that if I take it, and in the same quantity, at breakfast, I do not really digest it during the whole forenoon, and consequently have very little appetite, if any, for my dinner."

Doctor—"Madam, your experience is true. That is because one's stomach, so soon after rising in the morning, is not always in a condition to digest strong food. The whole system has been at rest during the night, and the digestive organs have not had time to resume their wonted activity by exercise of the body. The higher classes of Europe, and also the middle and lower classes to a great extent, almost universally eat upon these philosophic principles. Their breakfast is usually a cup of tea or coffee, a slice or two of cold bread, a little fresh butter with it, and perhaps two or three soft boiled eggs. Their supper is also generally of the same light character, dinner being really the only substantial meal they eat. and that should be the rule with every one who wishes to have good health, long life, a clear brain and cheerful spirits."

Mother—"How different are we Americans, as a rule, and especially we Southerners. It is meat, meat, and usually pork meat for breakfast, dinner and supper. No wonder we are a nation of sallow dyspeptics, of ailing men, women and children, with scarcely strength to walk a few blocks in the city without becoming fatigued. Even the delicate, tender stomachs of our children are stuffed with meat three times a day, and usually fried. I know ladies of position in society, who make their supper the largest meal of the day—also eat heartily of meat at the time, and it oftener hot than cold."

Doctor—"Do these ladies seem to have good health?"

Mother—"Well, I cannot really say, Doctor. Some of them have enough flesh, but it does not seem to be sound, healthy flesh, because they have no natural color—their complexions are not clear and bright like I think they would be with persons in good health. They are often complaining of biliousness and headache, and for relief dose themselves every night with bromide of potassium or morphine, and take purgative pills two or three times a week."

Doctor—"I am not surprised, madam, that these ladies, to whom you refer, do not at all times feel altogether well, strong and bright, with a dose of bromide of potassium or morphine every night to relieve the pain produced by an over-loaded stomach, and a dose of cathartic pills to relieve constipation produced by the bromide and morphine. How could one expect to be otherwise than bilious or sallow and swarthy-looking. Do you know, madam, that the word bilious, not being understood, has caused more deaths than anything known to the profession. No one understands its meaning, and hence the entire human family think they are bilious once or twice a week, whether they have too little or too much bile, and thousands shorten their lives by taking purgatives. I could give you a number of instances which came under my personal observation."

Mother—"I don't doubt it. I never dare to eat any kind of meat for supper, only sometimes in the winter season if I am very hungry at the hour for tea, and then nothing stronger than cold chicken, cold beef or stewed oysters. If I am in my usual health, it does not hurt me to eat of these dishes moderately at that time. But I must say, Doctor, that I am more and more convinced that the sin of continually violating the laws of health lieth at the door of the profession. We are ignorant of the simple laws of hygiene, and it really seems to be the policy of the profession to keep us so. This is a mistaken policy, and hurtful in principle. I have always believed it to be the solemn duty of every physician to teach his patrons how to prevent disease as well as to cure it, when called upon."

Doctor—"I fully agree with you, madam, but it can never be done until the people are made familiar with our duties to them, and their obligations to us; and thousands will continue to die prematurely, and our profession will never attain that point in the estimation of the people, to which its great intrinsic merits justly entitle it, until its members are taught to see and made to feel this great error."

Mother—"Yes, Doctor, this should be done, and could be done if the Doctors would talk to their patients and friends as you are now talking to me; but allow me to ask you, before I forget, why is it that we can eat more meat in cold weather and feel no ill effects from it than we can in summer?"

Doctor—"It is because heat produces a feebleness of the whole system, and consequently of the digestive organs. When these organs are in that condition, of course they cannot digest such strong food as fat pork, or much meat of any kind, neither vegetables smothered in grease. Such food produces much internal heat, and this excess of warmth is certainly not needed by the system in hot weather. Whereas, the colder the climate the more meat can be eaten without injury, especially at the dinner hour."

Mother—"Then in warm weather we should eat very moderately of even the milder meats. How about fruits and vegetables?"

Doctor.—"That depends upon circumstances. If the system should be at the time over-supplied with acids, fruits and many vegetables will be injurious, but if deficient in acids, sound and fully matured fruit, and also fresh, nutritious vegetables, especially those that can be eaten without cooking, as tomatoes, cold slaw, etc., may be allowed. When adapted to the wants of the system they are cooling, grateful and wholesome; the acids they contain are furnished from nature's own laboratory. I have a number of friends who are never so healthy as when they can get plenty of grapes, and others who suffer from acid stomach, nervous headache, indigestion and nettle rash frequently during the fruit season."

Mother.—"I see, Doctor. Indeed, it is the experience of every one that the diet that is suitable and nourishing to-day, either from some change in the food or in the condition of the system, may be to-morrow unsuitable and productive of much suffering, and in order to be healthy and keep so, the laws of every-day life must be understood and obeyed; and to do this every one must be made part of a doctor, and as I was not taught at school, I desire now, for the reason stated, to learn all I can. Please tell me what you think of warm, heavy suppers? Do you know that I have heard some persons say that when they have been taking a great deal of exercise during the day, they needed a hearty, full, warm supper to 'set them up.'"

Doctor—"Yes, madam, that you have heard all you say I have not the slightest doubt. I have frequently heard similar statements, and in many instances I am equally satisfied that if they were not 'set up' they were kept awake the most of the night. It is a mistaken idea that these kinds of suppers are needed by persons who have been greatly fatigued through the day. It is a violation of the laws of natural economy, or, as you have said, 'of every-day life,' for one when greatly fatigued to eat at all until the body has had time to rest, and then not immoderately, especially if at night. Every physician could give a few cases, and hundreds are reported in the journals, of persons who retired to bed in robust health and died before morning alone from the effects of a full, warm supper; and this is not the greatest and most distressing result of over-eating, especially at night."

Mother.—"Oh! Doctor! shocking! what could be worse?"

Doctor.—"Many things, madam. For instance, daily unnecessary bickerings between man and wife. Did you ever think that perhaps the stupid and irritable condition of mind that the wife or husband woke up in to begin the day with, and all that followed through the day, was often the result of over and unsuitable supping? I am aware that the world generally, and especially you, ladies whose digestion is good, and are amiable persons, believe these ladies and gentlemen who rise in the morning in this irritable state of mind are disappointed in their marriage, or are constitutionally bad people. But this is a mistake; often some of the best and purest of our race are made hateful to themselves, their family and friends by the effect of imprudent diet."

Mother.—"I believe all you say, and can see that thousands of men and women kill themselves slowly or suddenly by their ignorance of the laws of health, and often their own children too, when they would give all they possess to save them from pain or death. Doctor, you must continue to talk with me until I know all these things."

Doctor.—"It will afford me pleasure, but I must go; I must meet another engagement in a few moments."

Mother.—"I am sorry that you are compelled to leave. Excuse me for keeping you so long. You will come again soon, will you not?"

Doctor.—"Yes, madam, I will call again in a few days. I wish to watch your daughter's case closely until she is restored to health. Good morning."

